# Rectal artesunate for severe malaria, implementation research, Zambia

**DOI:** 10.2471/BLT.22.289181

**Published:** 2023-04-17

**Authors:** Cathy Green, Paula Quigley, Tendayi Kureya, Caroline Barber, Ernest Chanda, Busisiwe Moyo, Bernard Mpande, Kenneth Mubuyaeta, Mutinta Mudenda, Likando Mundia, Ruth Nyirenda, Auxilia Piringondo, Hans Rietveld, Sebastian Simpasa, Dennis Simuyuni, Garikai Zinumwe

**Affiliations:** a Transaid, 137 Euston Road, London NW1 2AA, England.; b Development Alternatives Incorporated, Global Health, London, England.; c Development Data, Lusaka, Zambia.; d MAM@Scale, Lusaka, Zambia.; e Disacare, Lusaka, Zambia.; f National Malaria Elimination Centre, Department of Public Health, Lusaka, Zambia.; g Medicines for Malaria Venture, Meyrin, Switzerland.

## Abstract

**Objective:**

To determine whether the positive results of a single-district pilot project focused on rectal artesunate administration at the community level in Zambia could be replicated on a larger scale.

**Methods:**

In partnership with government, in 10 rural districts during 2018–2021 we: (i) trained community health volunteers to administer rectal artesunate to children with suspected severe malaria and refer them to a health facility; (ii) supported communities to establish emergency transport, food banks and emergency savings to reduce referral delays; (iii) ensured adequate drug supplies; (iv) trained health workers to treat severe malaria with injectable artesunate; and (v) monitored severe malaria cases and associated deaths via surveys, health facility data and a community monitoring system.

**Results:**

Intervention communities accessed quality-assured rectal artesunate from trained community health volunteers, and follow-on treatment for severe malaria from health workers. Based on formal data from the health management information system, reported deaths from severe malaria reduced significantly from 3.1% (22/699; 95% confidence interval, CI: 2.0–4.2) to 0.5% (2/365; 95% CI: 0.0–1.1) in two demonstration districts, and from 6.2% (14/225; 95% CI: 3.6–8.8) to 0.6% (2/321; 95% CI: 0.0–1.3) in eight scale-up districts.

**Conclusion:**

Despite the effects of the coronavirus disease, our results confirmed that pre-referral rectal artesunate administered by community health volunteers can be an effective intervention for severe malaria among young children. Our results strengthen the case for wider expansion of the pre-referral treatment in Zambia and elsewhere when combined with supporting interventions.

## Résumé

**Objectif:**

Déterminer si les résultats positifs d'un projet pilote mené dans un seul district et axé sur l'administration d'artésunate rectal au niveau communautaire en Zambie pourraient être reproduits à plus grande échelle.

**Méthodes:**

Nous avons collaboré avec le gouvernement zambien dans dix districts ruraux entre 2018 et 2021 pour (i) former des volontaires de santé au sein des communautés afin qu'ils puissent administrer de l'artésunate rectal aux enfants souffrant potentiellement d'une forme aiguë de paludisme et les renvoyer vers un établissement de soins ; (ii) soutenir les communautés afin qu'elles puissent mettre en place un transport d'urgence, des banques alimentaires et un fonds d'urgence destinés à réduire les retards de transfert ; (iii) assurer un approvisionnement en médicaments adéquat ; (iv) former des professionnels de la santé au traitement du paludisme aigu à l'aide d'artésunate injectable ; et enfin, (v) surveiller les cas de paludisme aigu et les décès qui y sont associés par le biais d'enquêtes, de données issues des établissements de soins et d'un système de suivi communautaire.

**Résultats:**

Les communautés dans lesquelles nous sommes intervenus ont pu avoir accès à de l'artésunate rectal de qualité garantie, administré par des volontaires de la santé formés à cet effet, tandis que les formes aiguës de paludisme ont été prises en charge par des professionnels de la santé. D'après les données officielles provenant du système d'information pour la gestion sanitaire, le nombre de décès attribuables à un paludisme aigu a considérablement baissé, passant de 3,1 % (22/699 ; intervalle de confiance (IC) de 95 % : 2,0–4,2) à 0,5 % (2/365 ; IC de 95 % : 0,0–1,1) dans deux districts de démonstration, et de 6,2 % (14/225 ; IC de 95 % : 3,6-8,8) à 0,6 % (2/321 ; IC de 95 % : 0,0–1,3) dans huit districts à plus grande échelle.

**Conclusion:**

Malgré l'impact de la maladie à coronavirus, nos résultats ont confirmé que l'administration d'artésunate rectal avant transfert par des volontaires de la santé au sein des communautés pouvait se révéler efficace dans la prise en charge des formes aiguës de paludisme chez les jeunes enfants. Ces résultats plaident en faveur d'une extension du traitement avant transfert en Zambie et ailleurs lorsqu'il est associé à des interventions de soutien.

## Resumen

**Objetivo:**

Determinar si los resultados positivos de un proyecto piloto de un solo distrito centrado en la administración rectal de artesunato a nivel comunitario en Zambia se podrían reproducir a mayor escala.

**Métodos:**

En asociación con el gobierno, en 10 distritos rurales entre 2018 y 2021 (i) se capacitó a voluntarios sanitarios de la comunidad para administrar artesunato rectal a niños con sospecha de paludismo grave y derivarlos a un centro sanitario; (ii) se apoyó a las comunidades para establecer transporte de emergencia, bancos de alimentos y ahorros de emergencia para reducir las demoras en la derivación; (iii) se garantizaron suministros adecuados de medicamentos; (iv) se capacitó a trabajadores sanitarios para tratar el paludismo grave con artesunato inyectable; y (v) se monitorearon los casos de paludismo grave y las muertes asociadas mediante encuestas, datos de centros sanitarios y un sistema de monitoreo comunitario.

**Resultados:**

Los voluntarios sanitarios de la comunidad formados administraron artesunato rectal de calidad garantizada a las comunidades de intervención, y los trabajadores sanitarios administraron tratamiento de seguimiento para el paludismo grave. Según los datos formales del sistema de información de gestión sanitaria, las muertes por paludismo grave se redujeron significativamente del 3,1% (22/699; intervalo de confianza del 95%: 2,0-4,2) al 0,5% (2/365; IC del 95%: 0,0-1,1) en dos distritos de demostración y del 6,2% (14/225; IC del 95%: 3,6-8,8) al 0,6% (2/321; IC del 95%: 0,0-1,3) en ocho distritos de ampliación.

**Conclusión:**

A pesar de los efectos de la enfermedad por coronavirus, los resultados confirmaron que el artesunato rectal administrado antes de la remisión por voluntarios sanitarios de la comunidad puede ser una intervención eficaz contra el paludismo grave en niños pequeños. Estos resultados refuerzan los argumentos a favor de una mayor expansión del tratamiento prerreferencial en Zambia y en otros lugares cuando se combina con intervenciones de apoyo.

## ملخص

**الغرض:**

تحديد ما إذا كانت النتائج الإيجابية للمشروع التجريبي لمنطقة واحدة، والتي ركزت على التعامل مع أرتيسونات المستقيم على مستوى المجتمع المحلي في زامبيا، يمكن تكرارها على نطاق أوسع.

**الطريقة:**

بالشراكة مع الحكومة، وفي 10 مناطق ريفية خلال الفترة من 2018 إلى 2021، قمنا (1) بتدريب المتطوعين في مجال الصحة المجتمعية على التعامل مع أرتيسونات المستقيم لدى الأطفال الذين يشتبه في وجود إصابة حادة لديهم بالملاريا، وإحالتهم إلى مرفق صحي؛ و(2) دعم المجتمعات المحلية للإعداد للنقل في حالات الطوارئ، وإنشاء بنوك الطعام، وجمع الأموال في حالات الطوارئ لتقليل حالات التأخير في الإحالة؛ و(3) تأمين الإمدادات الكافية من الأدوية؛ و(4) تدريب العاملين في مجال الصحة على علاج حالات الملاريا الحادة باستخدام مادة أرتيسونات القابلة للحقن؛ و(5) رصد حالات الملاريا الحادة والوفيات المرتبطة بها، من خلال المسوحات، وبيانات المرافق الصحية، ونظام الرصد المجتمعي.

**النتائج:**

حصلت مجتمعات التدخل على أرتيسونات المستقيم مضمونة الجودة من المتطوعين الذين تم تدريبهم في مجال الصحة المجتمعية، ومتابعة علاج الملاريا الحادة من العاملين في المجال الصحي. استنادًا إلى البيانات الرسمية من نظام معلومات الإدارة الصحية، انخفضت حالات الوفيات المّبلغ عنها من الملاريا الحادة بشكل ملحوظ من %3.1 (22/699؛ بنسبة أرجحية مقدارها %95: 2.0 إلى 4.2) إلى %0.5 (2/365 بنسبة أرجحية مقدارها %95: 0.0 إلى 1.1) في منطقتين من مناطق التنفيذ ومن %6.2 (14/225؛ بنسبة أرجحية مقدارها %95: 3.6 إلى 8.8) إلى %0.6 (2/321 بنسبة أرجحية مقدارها %95: 0.0 إلى 1.3) في ثماني مناطق واسعة النطاق.

**الاستنتاج:**

على الرغم من آثار مرض فيروس كورونا، أكدت نتائجنا أن أرتيسونات المستقيم قبل الإحالة، والتي قام بالتعامل معها المتطوعون في مجال الصحة المجتمعية، يمكن أن تكون تدخلاً فعالاً لحالات الملاريا الحادة بين الأطفال الصغار. تدعم نتائجنا حالة التوسع الأشمل لعلاج ما قبل الإحالة في زامبيا وأماكن أخرى عندما يقترن ذلك بالتدخلات الداعمة.

## 摘要

**目的:**

旨在确定是否可以大规模地复制赞比亚社区层面以青蒿琥酯直肠给药为重点的单一地区试点项目的积极成果。

**方法:**

2018 至 2021 年期间，我们与政府合作，在 10 个农村地区 (i) 培训社区卫生志愿者，为疑似患有重症疟疾的儿童直肠给药青蒿琥酯，并将他们转诊到医疗机构；(ii) 支持社区建立紧急运输、食物赈济处和应急储蓄，以减少转诊延误；(iii) 确保充足的药品供应；(iv) 培训卫生工作者使用注射用青蒿琥酯治疗重症疟疾；并且 (v) 通过调查以及利用卫生设施数据和社区监测系统监测重症疟疾病例和相关死亡率。

**结果:**

在干预社区，先由接受过培训的社区卫生志愿者有效实施青蒿琥酯直肠给药，然后由卫生工作者为重症疟疾患者进行后续治疗。根据卫生管理信息系统提供的官方数据，两个示范区报告的重症疟疾病例死亡人数已从 3.1%（22/699；95% 置信区间，CI：2.0-4.2）显著下降至 0.5%（2/365；95% CI：0.0-1.1），且八个扩大规模地区报告的对应死亡人数也已从 6.2%（14/225；95% CI3.6-8.8）显著下降至 0.6%（2/321；95% CI：0.0-1.3）。

**结论:**

尽管受到冠状病毒疾病的影响，但我们的研究结果证实，社区卫生志愿者在患者转诊前直肠给药青蒿琥酯可以有效降低幼儿的重症疟疾患病率。我们的研究结果进一步证明，可在赞比亚和其他地方结合支持性干预措施扩大转诊前治疗规模。

## Резюме

**Цель:**

Определить, могут ли положительные результаты экспериментального проекта в одном районе Замбии по введению ректальных свечей, содержащих артесунат, на местном уровне быть воспроизведены в более широком масштабе.

**Методы:**

В партнерстве с правительством в 10 сельских округах в течение 2018–2021 годов: (i) было проведено обучение волонтеров, обеспечивающих медицинское обслуживание местного населения, практике введения ректальных свечей, содержащих артесунат, детям с подозрением на тяжелую форму малярии с последующим направлением таких детей в медицинские учреждения; (ii) оказана поддержка общинам в обеспечении аварийно-спасательным транспортом, создании банков продовольствия и сбережений на случай чрезвычайных ситуаций для сокращения задержек с направлением в медицинские учреждения; (iii) были обеспечены необходимые поставки лекарств; (iv) проведено обучение медицинских работников методам лечения тяжелой формы малярии с помощью артесуната для инъекций; (v) проведен мониторинг случаев тяжелой формы малярии и связанных с ней смертей с помощью опросов, данных медицинских учреждений и системы мониторинга на местном уровне.

**Результаты:**

Общины, участвующие в программе, получили доступ к ректальным свечам, содержащим артесунат гарантированного качества, которые применяли обученные волонтеры, обеспечивающие медицинское обслуживание местного населения, и к последующему лечению тяжелой формы малярии медицинскими работниками. Согласно официальным данным из информационной системы управления в области здравоохранения, зарегистрированные случаи смерти от тяжелой формы малярии значительно снизились с 3,1% (22/699; 95%-й ДИ: 2,0–4,2) до 0,5% (2/365; 95%-й ДИ: 0,0–1,1) в двух демонстрационных районах и с 6,2% (14/225; 95%-й ДИ: 3,6–8,8) до 0,6% (2/321; 95%-й ДИ: 0,0–1,3) в восьми районах расширения масштаба.

**Вывод:**

Несмотря на последствия коронавирусного заболевания, полученные результаты подтвердили, что применение волонтерами, обеспечивающими медицинское обслуживание местного населения, ректальных свечей, содержащих артесунат, может быть эффективным средством борьбы с тяжелой формой малярии среди детей раннего возраста. Эти результаты подтверждают необходимость более широкого распространения добольничного лечения в Замбии и других странах в сочетании с поддерживающими мероприятиями.

## Introduction

Malaria deaths reduced by over one third in sub-Saharan African countries during 2010–2019, although the rate of reduction began to fall during the second half of that period. Globally, malaria deaths increased by 69 000 to 627 000 between 2019 and 2020. An estimated 47 000 of the additional deaths were the result of disruptions to malaria services caused by the coronavirus disease 2019 (COVID-19) pandemic.^1^ The World Health Organization’s (WHO) global malaria strategy milestone for 2020 was not reached. Zambia experienced a 11.9% increase in malaria mortality from 2019 to 2020, with an estimated 7996 deaths in 2019 and 8946 in 2020. An estimated 80% of these deaths were among children younger than 5 years.^1^ Disruptions to service delivery and delayed access to services contributed to the upsurge in mortality.

In rural communities in Zambia, multiple barriers delay the referral of sick children to a health facility. Uncomplicated malaria can quickly turn into severe malaria if not treated, resulting in high mortality (60–100% of cases),^2^^,^^3^ especially among children younger than 5 years. The administration of rectal artesunate capsules at the community level before referral to a health facility has the potential to significantly reduce child deaths from severe malaria. The efficacy and safety of rectal artesunate has been confirmed in various studies.^4^^–^^6^ The drug helps to stabilize children, giving families who live far from a health facility vital time to implement a referral plan.

Rectal artesunate was included in Zambia’s national malaria guidelines in 2014 and introduced at the community level in 2017. Until 2017, injectable artesunate – the first-line drug for treatment of severe malaria – was only available in district and tertiary hospitals.

In our July 2017–July 2018 pilot intervention in Zambia, we trained community health workers to administer rectal artesunate to children with suspected severe malaria;^7^ our results indicated a reduction in reported severe malaria case fatalities from 8.0% (18/224) in 2017 to 0.5% (3/619) in 2018. We subsequently aimed to explore whether these results could be replicated on a larger scale in other rural areas of high malaria burden. We established MAMaZ Against Malaria at Scale (MAM@Scale) in December 2018, a follow-up project to the pilot intervention, and implemented this intervention in 10 rural districts over 3 years. We provide results and analysis to demonstrate the extent to which the intervention increased the access of rural communities to effective treatment for severe malaria.

## Methods

### Study design

We conducted an observational, cross-sectional study using quantitative data from: (i) surveys of community health volunteers and participating health facilities at the beginning, middle and end of the intervention; (ii) a community monitoring system; and (iii) two verification studies that explored whether cases of severe malaria had been managed correctly according to WHO protocols.^8^

### Intervention sites

**Fig. 1:**
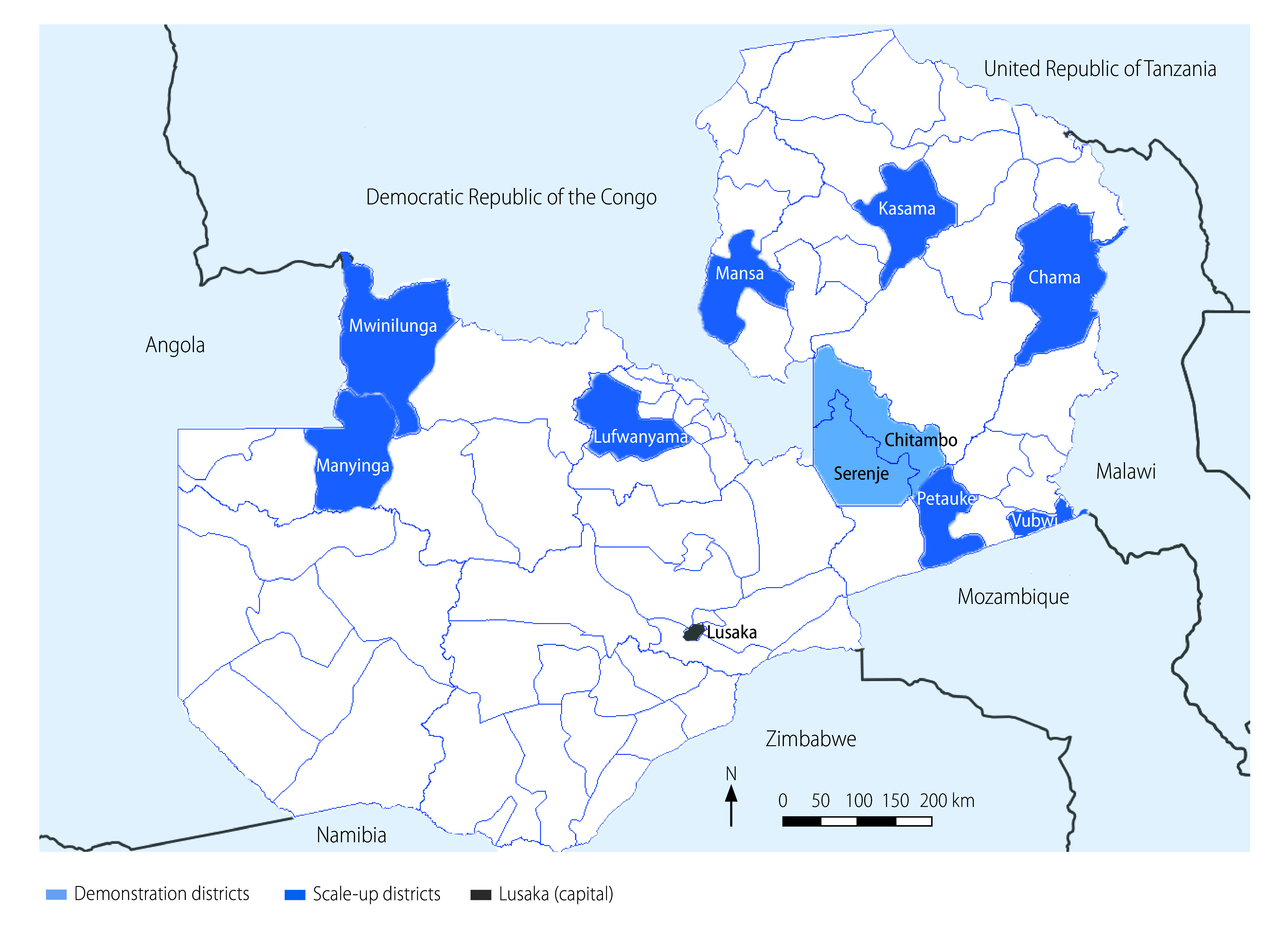
Districts included in implementation on use of rectal artesunate for severe malaria, Zambia 2018–2021

The health ministry in Zambia selected 10 rural intervention districts, all defined by the National Malaria Elimination Centre as having a high malaria burden (Table 1, available at https://www.who.int/publications/journals/bulletin/; Fig. 1). Our implementation began in December 2018 in Serenje (the site of the pilot intervention) and Chitambo; these demonstration districts hosted visits by health officials interested in scaling up the use of rectal artesunate. We phased in the implementation in eight other districts (scale-up districts) between July 2019 and February 2021, making the pre-treatment accessible to a total population of approximately 900 000. Over 200 (*n* = 217) lower-level health facilities (i.e. rural health centres and health posts) participated in the project. Intervention sites (*n* = 1272; Table 2), known as neighbourhood health committees, were located within the rural catchment areas of the health facilities.

**Table 1:** Districts selected for participation in implementation on use of rectal artesunate for severe malaria, Zambia, 2018–2021

District (province)	Estimated population coverage of intervention/district population^a^ (%)	No. intervention health facilities/total no. district health facilities (%)	Start of implementation
**Demonstration districts**			
Chitambo (Central)	52 000/61 348 (84.8)	14/14 (100.0)	Dec 2018
Serenje (Central)	108 000/148 006 (73.0)	25/29 (86.2)	Dec 2018
**Scale-up districts**			
Chama (Muchinga)	86 500/151 428 (57.1)	18/29 (62.1)	Jul 2019
Manyinga (North Western)	58 500/58 500 (100.0)	11/11 (100.0)	Jul 2019
Petauke (Eastern)	105 500/306 681 (34.4)	22/31 (71.0)	Nov 2019
Vubwi (Eastern)	35 000/55 015 (63.6)	12/12 (100.0)	Dec 2019
Mansa (Luapula)	108 000/253 414 (42.6)	37/74 (50.0)	Nov 2020
Kasama (Northern)	105 500/306 423 (34.4)	28/43 (65.1)	Nov 2020
Mwinilunga (North Western)	106 000/129 523 (81.8)	27/28 (96.4)	Nov 2020
Lufwanyama (Copper Belt)	133 500/150 000 (89.0)	23/23 (100.0)	Feb 2021

^a^ Data from Population and Housing Census 2010,^9^ adjusted for population growth and boundary changes.

**Table 2:** Properties of high- and low-intensity intervention sites^a^ participating in implementation on use of rectal artesunate for severe malaria, Zambia, 2018–2021

Component	No. (%)	
	High-intensity support	Low-intensity support
Sites	158/1272 (12.4)	1114/1272 (87.6)
Sites receiving particular support	In demonstration districts: 158/158 (100.0)	In demonstration districts: 22/1114 (2.0); in scale-up districts: 1 092/1114 (98.0)
Training focus	Rectal artesunate administration; other child health issues (uncomplicated malaria, pneumonia and severe diarrhoea); community mobilization	Rectal artesunate administration; integrated community case management; community mobilization
Training duration	3 days	2 days (less training in community mobilization than for high-intensity support)
Volunteers trained	Minimum 5 per site	1 per site
Volunteers trained /total community health volunteers	1500/3251 (46.1)	1751/3251 (53.9)
Volunteer to population ratio	1:250	1:500 upwards
Volunteers targeted	Safe motherhood action group volunteers and integrated case management volunteers	Integrated case management volunteers
Supervision	Local health facility; ongoing mentoring support from project	Local health facility; occasional mentoring support from project
Community systems (food banks and emergency savings schemes)	Active support provided to communities to establish these systems	Volunteers learnt how to establish these systems, but limited follow-up support provided
Bicycle ambulances/total available	100/144 (69.4)	44/144 (30.6)

^a^ Intervention sites (also known as neighbourhood health committees) were located in the rural catchment areas of the 217 health facilities mentioned in Table 1.

We provided different levels of support to the intervention sites: 12.4% (158) of these sites, mainly located within the demonstration districts, received higher-intensity support. The eight scale-up districts were supported with inputs that were conceivably closer to real-world conditions. The main differences between high- and low-intensity support were in volunteer to population ratios, duration of volunteer training, extent of supportive supervision and level of support given to community systems (Table 2). District partnership agreements, signed by the district health teams, set out how the intervention would be mainstreamed into the routine activities of government.

### Training

We instigated a large-scale training programme (Table 3). With the district health team, we trained community health volunteers with prior training in safe motherhood or integrated community case management to administer rectal artesunate to children showing signs of severe malaria, and refer them to a health facility. Signs included fever in combination with one or more of: seizures, lethargy or unconsciousness, vomiting everything, or inability to eat or drink. District trainers trained community health volunteers, who then trained their communities. This approach helped build local capacity to sustain the intervention.

**Table 3:** Health workers and community health volunteers trained during implementation on use of rectal artesunate for severe malaria, Zambia, 2018–2021

Type of training	Target audience	No. trained (%)	No. districts
Rectal artesunate administration and community mobilization	Community health volunteers (*n* = 3 251)	2301 (70.8)	All 10
Community mobilization only	Community health volunteers (*n* = 3 251)	950 (29.2)	2: demonstration districts
Emergency transport scheme management and operation	Community volunteer riders and custodians (*n* = 343)	343 (100.0)	4: demonstration districts, Vubwi and Chama
Severe malaria case management	Health workers (clinical officers and nurses; *n* = 726)	726 (100.0)	All 10

In the demonstration districts, some community health volunteers received a 3-day stand-alone training course in rectal artesunate and community mobilization, while others received training in community mobilization only. In the scale-up districts, district trainers delivered a 2-day training course as part of the national roll-out of integrated community case management training. The volunteers were unpaid, in line with national policy, and conducted their severe malaria activities alongside other voluntary activities.

Volunteers also received training in facilitating community discussion groups and undertaking door-to-door visits, both of which were used to raise awareness of the danger signs of severe malaria and the need for prompt action. They worked with communities to establish community-managed systems to address referral delays (e.g. food banks and emergency savings schemes). In addition, volunteers received training in the identification and inclusion of socially excluded and vulnerable individuals in project activities. Volunteers were encouraged to look beyond poverty as a cause for exclusion, and to focus on other contributing factors such as sex- and gender-based violence, mental health or disability.^10^

### Emergency transport

In demonstration communities, we extended and strengthened emergency transport systems (i.e. robust bicycles and covered trailers that were suitable for the terrain) that had been established in earlier projects.^11^^,^^12^ We also provided two scale-up districts (Chama and Vubwi) with new bicycle ambulances and placed these in locations lacking alternative transport options. We trained riders to transfer patients safely to the health facility; riders were not requested to administer drugs, so received no training in this area. 

### Implementation

In 2019 we procured two batches of 100 mg quality-assured rectal artesunate, and later that year the health ministry started to procure the drug. We made the treatment available in all project areas, and trained volunteers delivered a rapid diagnostic test for malaria if symptoms were observed, and administered rectal artesunate in line with WHO recommendations.^8^ We provided refresher training in severe malaria and injectable artesunate administration to clinical officers and nurses in rural health centres and health posts. The National Malaria Elimination Centre worked closely with the districts to ensure reliable supplies of injectable artesunate were available through the routine drug supply system. Health facility staff supervised volunteers, including bicycle ambulance riders, during community outreach sessions or at the health facility. District health teams retained oversight of volunteer supervision in their routine activities. Volunteers were trained to follow up discharged patients (within a few hours of their return and then once a week for up to a month) and observe them for potential side-effects.

Trained volunteers recorded data on their activities in a community monitoring system that was shared with the health facility for onward transmission to the district health office. The community monitoring system generated data on simple and severe malaria cases; rapid diagnostic test use and results; use of referral and counter-referral forms; beneficiaries of community systems; number of follow-up visits; and number of child deaths from malaria (Box 1). Volunteers routinely recorded indicators for a particular section of the community. Lead volunteers aggregated the data at the community level, having reviewed and collated reports generated by colleagues. Data review by community health volunteers and district health offices informed decision-making on key issues, for example, where to focus volunteer efforts to ensure all parts of the community were reached. The joint planning that took place following data review contributed to the motivation and retention of volunteers.

Box 1 **Data collected by community health volunteers and riders during implementation on use of rectal artesunate for severe malaria, Zambia, 2018–2021**

1. Uncomplicated malaria cases
2. Suspected severe malaria cases
3. tested with a rapid diagnostic test for malaria
4. Positive rapid diagnostic tests
5. Rectal artesunate recipients
6. referred to a health facility by a community health volunteer
7. received a counter-referral form from health facility
8. followed up by volunteers
9. Children with suspected severe malaria
10. supported by emergency transport system
11. supported by food banks
12. supported by savings schemes
13. who died
14. Discussion group sessions held in the community
15. Door-to-door visits undertaken by community health workers.

Note: Zambia’s health management information system did not include a community component during the timeframe of the project. Data collected at community level were therefore specific to the project. Community health volunteers reported data to the local health facility for onward transmission to the district health management team, where they were used for ongoing management decision-making.

District trainers provided a workshop on COVID-19 in April 2020 to enable the volunteers to undertake preventive activities and ensure an appropriate response to cases. Community health volunteers encouraged their communities to adapt food banks and savings schemes to support families affected by COVID-19. Bicycle ambulance riders received training on safe operation within the context of the pandemic.

### Data management

Our main outcome indicators were proportion of children with suspected severe malaria who received rectal artesunate in the community, and proportion of children given rectal artesunate who were referred to a health facility. The main impact indicator was reduction in severe malaria case fatalities. Of the three surveys undertaken at the beginning (January 2019), middle (July 2020) and end (October 2021) of the implementation, the first provided a baseline for the two demonstration districts, the second assessed progress in the demonstration districts and served as a baseline for the scale-up districts, and the third assessed progress in five districts. 

We developed two assessment tools: a facility audit tool assessed human resources, drug supplies and severe malaria case management practices; and a semi-structured interview tool collected data from volunteers and bicycle ambulance riders. A total of 22.1% (48) and 18.4% (40) of all intervention health facilities participated in the middle and end surveys, respectively. The end survey included feedback from 15.1% (544/3594) of the trained volunteers and riders. Independent trained enumerators collected survey data using tablet computers. We provided no financial incentives to interview respondents to participate in the surveys. All quantitative data submitted to the survey server were downloaded as comma-separated values (csv) text files and analysed using SPSS version 25 (SPSS Inc., Chicago, United States of America). Statistical significance was assessed at *P* < 0.05 (two-sided).

We collected data on total malaria cases, as well as the project-specific indicators of numbers of severe malaria cases (in children aged < 1 year and 1–5 years) and deaths, from health facilities. Volunteers and riders collected data on 14 indicators (Box 1). In collaboration with the district health teams of the demonstration districts, we led two verification studies in October 2019 and December 2020 to assess whether volunteers were correctly following WHO rectal artesunate protocols (Box 2). By combining staff from different areas of the implementation project we reduced any risk of bias. The 10 health facilities selected for the verification study had reported a high number of suspected severe malaria cases. In each study, 10 randomly selected cases of suspected severe malaria per health facility were examined, meaning that 100 cases were reviewed. The study team worked with health facility staff to establish whether patients given rectal artesunate in the community had received a rapid diagnostic test for malaria, the result of the test, whether the patients had been treated for severe malaria at the health facility, what malaria drugs were administered and whether the patient survived.

Box 2 **Data collected in verification studies on use of rectal artesunate for severe malaria, Zambia, 2018–2021**

1. Age of patient
2. Sex of patient
3. Date of arrival at health facility
4. Location at which rectal artesunate administered
5. Result of rapid diagnostic test for malaria
6. Location of rapid diagnostic test for malaria
7. Severe malaria danger signs recognized by community health volunteer and/or child’s caregiver
8. Time taken (in minutes) to get to the health facility after child given rectal artesunate
9. Whether child received treatment for severe malaria at the health facility
10. Name of antimalarial drug(s) given at the health facility
11. Reason rectal artesunate recipient was not given injectable artesunate
12. Patient outcome

### Ethical considerations

The research was covered by a protocol approved in August 2017 by the independent Excellence, Research, Ethics and Science (ERES) Converge ethical review board and the National Health Research Authority. We obtained written informed consent from all participants to use the data collected.

## Results

Malaria drug supplies improved in intervention health facilities over the timeframe of the project, with 35.3% (6/17; 95% confidence interval, CI: 12.5–57.9) of surveyed health facilities in the demonstration districts reporting stocks of the four main malaria drugs (i.e. sulfadoxine-pyrimethamine, quinine, injectable artesunate and artemisinin-based combination therapy) at baseline and 81.3% (13/16; 95% CI: 62.2–100.0) at the end. We observed an even greater improvement in the scale-up districts from 4.5% (1/22; 95% CI: 0.0–13.2) at scale-up baseline (i.e. middle survey) to 79.2% (19/24; 95% CI: 63.0–95.4) at the end (Table 4). In the end survey, 92.3% (60/65; 95% CI: 62.7–95.3) of health workers in all surveyed facilities who had been trained in injectable artesunate reported managing cases of severe malaria.

**Table 4:** Key results of implementation on use of rectal artesunate for severe malaria, disaggregated by district type, Zambia, 2018–2021

Indicator	Surveyed health facilities, health workers or volunteers								
	Demonstration districts					Scale-up districts			
	Baseline (Jan 2019)		End (Oct 2021)			Middle (Jul 2020)		End (Oct 2021)	
	No. (total)	% (95% CI)	No. (total)	% (95% CI)		No. (total)	% (95% CI)	No. (total)	% (95% CI)
Health facilities reporting stocks of four main malaria drugs	6 (17)	35.3 (12.5–57.9)	13 (16)	81.3 (62.2–100.0)		1 (22)	4.5 (0.0–13.2)	19 (24)	79.2 (63.0–95.4)
Health workers trained in injectable artesunate who reported that they had managed cases of severe malaria	NA	NA	28 (29)	96.6 (93.0–100.0)		NA	NA	22/26	85 (63.0–100.0)
Community health volunteer knowledge of five severe malaria danger signs	37 (421)	8.8 (4.7–12.9)	173 (224)	77.2 (68.5–86.1)		59 (187)	31.6 (26.5–36.7)	222 (315)	70.5 (44.8–84.0)
Community health volunteers who had ever managed a case of severe malaria	337 (426)	79.1 (73.4–83.0)	213 (225)	94.7 (91.8–97.6)		168 (188)	89.4 (85.8–92.6)	299 (319)	93.7 (91.0–96.4)
Community health volunteers who reported that they were confident to administer rectal artesunate	91 (213)	42.7 (16.3–65.2)	225 (225)	100.0 (NA)		176 (185)	95.1 (92.4–100.0)	317 (319)	99.4 (98.0–100.0)
Community health volunteers reporting that they had taken actions to include the least-supported women in project activities	NA	NA	212 (225)	94.2 (91.3–97.3)		NA	NA	277 (319)	86.8 (83.0–90.8)

CI: confidence interval; NA: not applicable.

Knowledge among community health volunteers of the five severe malaria danger signs improved both in demonstration districts, from 8.8% (37/421; 95% CI: 4.7–12.9) to 77.2% (173/224; 95% CI: 68.5–86.1), and in scale-up districts, from 31.6% (59/187; 95% CI: 26.5–36.7) to 70.5% (222/315; 95% CI: 44.8–84.0). Over the project timeframe, the proportion of volunteers who said that they had ever managed a case of severe malaria increased from 79.1% (337/426; 95% CI: 73.4–83.0) to 94.7% (213/225; 95% CI: 91.8–97.6) in the demonstration districts, and from 89.4% (168/188; 95% CI: 85.8–92.6) to 93.7% (299/319; 95% CI: 91.0–96.4) in the scale-up districts (Table 4). In the end survey, 99.6% (542/544; 95% CI: 99.1–100.0) of responding volunteers reported that they were confident in the administration of rectal artesunate.

Volunteers conducted almost 153 000 door-to-door visits and facilitated more than 91 000 community discussion groups. In 2020, new national COVID-19 guidelines for community health workers specified that household visits could be undertaken if social distancing protocols were followed. During intermittent periods of lockdown in 2020, three quarters of the volunteers were forced to curtail their mobilization activities. Nevertheless, community monitoring system data indicated that they remained active and made positive efforts to reach and involve entire communities in awareness-raising activities. In the end survey, 90.1% (490/544; 95% CI: 87.5–92.5) of responding volunteers reported having taken actions to include the least-supported women in project activities.

Data reported through the community monitoring system indicated that of the 11 486 children identified with suspected severe malaria at community level, 96.6% (11 095/11 486) were given rectal artesunate. Rapid diagnostic tests were given in 96.2% (11 054/11 486) of identified cases (Table 5). The community monitoring system established that almost all children given rectal artesunate (99.7%; 11 057/11 095) were referred to a health facility and, in turn, 83.0% (9 207/11 095) received a counter-referral form (Table 5). Based on data from the community monitoring system, 91.4% (10 139/11 095) of rectal artesunate recipients were followed up at least once. Over half (52.0%, 283/544) of the volunteers who participated in the end survey followed up patients three or more times, correctly following protocol. 

**Table 5:** Key results from community monitoring system of children with suspected severe malaria given rectal artesunate in demonstration and scale-up districts, Zambia, 2018–2021

Children	No. (%)		
	Demonstration districts	Scale-up districts	All districts
**With suspected severe malaria**	5 896 (100.0)	5 590 (100.0)	11 486 (100.0)
Given rectal artesunate at community level	5 809 (98.5)	5 286 (94.6)	11 095 (96.6)
Given rapid diagnostic tests	5 674 (96.2)	5 380 (96.2)	11 054 (96.2)
**Of those given rectal artesunate**			
Referred to health facility	5 778 (99.5)	5 279 (99.9)	11 057 (99.7)
Given a counter-referral form	4 393 (75.6)	4 814 (91.1)	9 207 (83.0)
Followed up at least once	5 051 (87.0)	5 088 (96.3)	10 139 (91.4)
Severe malaria referrals by bicycle ambulance	1 969 (33.9)	268 (5.1)	2 237 (20.2)
Food bank recipients	3 035 (52.2)	587 (11.1)	3 622 (32.6)
Emergency saving scheme recipients	2 700 (46.5)	858 (16.2)	3 558 (32.1)

Verification studies undertaken in the demonstration districts in 2019 and 2020 confirmed that 86.0% (86/100; 95% CI: 79.1–92.8) and 96.0% (96/100; 95% CI: 92.2–99.8) of suspected severe malaria cases referred from the community were given artemisinin-based combination therapy at the health facility, respectively; however, the proportion of cases who received injectable artesunate fell from 90.0% (90/100; 95% CI: 84.1–95.9) in 2019 to 63.0% (63/100; 95% CI: 53.5–72.5) in 2020. The community activities in 2020 took place against a backdrop of disruptions to health service delivery caused by COVID-19: in the July 2020 survey, 84.1% (37/44) of intervention facilities reported disruptions to medicines and consumables, and 61.4% (27/44) reported that they had suspended or scaled down community-level activities, which affected their volunteer supervision activities. By October 2021 the situation had improved; 45.0% (18/40) and 50.0% (20/40) of the surveyed health facilities reported disruptions in supplies and activities, respectively. 

The community-managed emergency savings schemes and food banks, which helped to increase affordability and overcome other practical constraints to referral, were well used. One third of patients’ families were supported by these schemes. Overall, 20.2% (2 237/11 095) of rectal artesunate recipients were transported to the health facility by bicycle ambulance, helping to reduce referral delays. A higher proportion of recipients benefited from emergency transport in the demonstration districts where there were more bicycle ambulances than in the scale-up districts: 33.9% (1969/5809) of cases versus 5.1% (268/5286; Table 5).

In the end survey, we collected health facility data on the number of deaths among children younger than 5 years over a 12-month period (September 2020 to August 2021) and compared these with data from the baseline and middle surveys (Table 6). In demonstration districts, there were two deaths recorded from 365 confirmed severe malaria cases, indicating a case fatality rate of 0.5% (95% CI: 0.0–1.1), down from 3.1% (22/699; 95% CI: 2.0–4.2) at baseline. In scale-up districts, two deaths were recorded from 321 cases, suggesting a case fatality rate of 0.6% (95% CI: 0.0–1.3), down from 6.2% (14/225; 95% CI: 3.6–8.8) from the middle survey. These results continued the trend of decreasing reported deaths from severe malaria observed in the pilot project.^7^

**Table 6:** Numbers of severe malaria cases, deaths and case fatality percentages during implementation on use of rectal artesunate for severe malaria, Zambia, 2018–2021

Survey	Demonstration districts			Scale-up districts		
	No. severe malaria cases	No. deaths	Case fatality percentage (95% CI)	No. severe malaria cases	No. deaths	Case fatality percentage (95% CI)
Baseline	699	22	3.1 (2.0–4.2)	NA	NA	NA
Middle	1032	10	1.0 (0.5–1.5)	225	14	6.2 (3.6–8.8)
End	365	2	0.5 (0.0–1.1)	321	2	0.6 (0.0–1.3)

CI: confidence interval; NA: not applicable.

## Discussion

Our results show that, in all 10 intervention districts, almost all children identified with suspected severe malaria in the community received rectal artesunate and were subsequently referred to a health facility. Our verification studies conducted in the demonstration districts confirmed that a high percentage of patients received quality care. Replicating these studies in the scale-up districts would provide important information on the quality of severe malaria case management in a wider scenario. Although volunteers in all districts were confident that all potential cases of severe malaria were identified, high population to volunteer ratios in the scale-up districts mean that it is possible that some cases were missed.

Unsurprisingly, the impact of the project was greater in the demonstration districts where we introduced the intervention earlier and provided higher-intensity support, and there had been a decade-long history of similar interventions.^12^ The food banks and emergency savings schemes supported almost five times the number of clients per month in these districts compared with the scale-up districts. Additional longitudinal studies would confirm the added value of the supplementary interventions in helping to reduce referral delays, and in sustaining demand for severe malaria services.

To ensure sustainability, the severe malaria activities were integrated into the delivery of routine health services. In the scale-up districts, rectal artesunate training was provided as part of integrated community case management training using an adapted version of the national training manual. District health teams led the training of community health volunteers and health staff; these personnel can be drawn on to support further scale-up into new districts. Volunteers were supervised by health facility staff, with district health teams providing oversight. Drug supplies and consumables were procured by the Zambia Medicines and Medical Supplies Agency and distributed through the essential drugs system. The health ministry procured rectal artesunate from 2019. Although district health teams had oversight of the community-managed emergency transport systems, they drew heavily on external technical support to fulfil this role; progress towards embedding oversight of these systems into routine activities was therefore slow.

In contrast to our results, the Community Access to Rectal Artesunate for Malaria project, implemented in 2018–2021 in the Democratic Republic of the Congo, Nigeria and Uganda, reported a negative impact on referral and case fatality numbers following deployment of rectal artesunate.^13^^,^^14^ However, several differences in study design – highlighting the strengths of our study – may have contributed to the different outcomes. First, in Zambia, the emphasis of the health ministry on building the capacity of health posts and rural health centres to detect, manage and refer severe malaria cases helped bring services closer to communities facing logistical, financial and other practical challenges when confronted with a child health emergency. In the multicountry study, the referral process was more complex in that rectal artesunate was administered either in the community or in primary health-care facilities, and follow-on treatment was provided at higher-level referral facilities. Second, although both interventions contained an awareness-raising component for communities, we addressed additional social determinants of health in Zambia: social exclusion, lack of transport, and lack of food and money. Transport systems halved travel times, and were available to the community at all times at no cost. The emphasis on social inclusion was important since these are often the families that carry the highest burden of child mortality.^15^^,^^16^ Third, our Zambia intervention allowed capacity for practical problem-solving to be built within district health teams, helping to identify solutions to health systems challenges. For example, when injectable artesunate supplies were affected by the pandemic in 2020, districts reported that the early notification of impending drug shortages using WhatsApp allowed supplies to be moved between facilities as needed. Fourth, in the multicountry study, rectal artesunate and injectable artesunate were frequently administered as monotherapies, suggesting that health worker training was deficient or that there were problems with drug supplies. In Zambia, our verification studies highlighted that health workers followed severe malaria protocols, including drug regimens, in almost all cases. Fifth, differences in project design may have affected the accessibility of rectal artesunate. In Zambia, we gave volunteers 10–20 capsules at any one time and advised them to collect further supplies from the local health facility. In the multicountry study, volunteers were allocated two capsules and required to travel to the health facility more frequently. This approach may have encouraged volunteers to prioritize more severe cases. Finally, based on the available evidence, it is probable that volunteers were supervised more regularly and consistently in Zambia. These engagements provided opportunities for implementation challenges to be identified and addressed, helping to improve outcomes.

The multicountry study results prompted WHO to issue additional guidance to countries planning to introduce or scale-up rectal artesunate in 2022.^17^ Current advice is to withhold new implementation or cease further expansion unless countries are able to review and verify that the conditions under which rectal artesunate is being used are appropriate regarding diagnosis, immediate referral and completion of treatment. This guidance has generated much debate.^18^^–^^21^ Zambia’s health ministry began scaling up rectal artesunate in 26 additional districts in 2021, and interpreted the WHO guidance as a call to proactively monitor and strengthen the quality of severe malaria case management at health facilities.

We have demonstrated that rectal artesunate administered at community level by trained volunteers, in combination with improvements in the continuum of care, can help reduce malaria-related mortality among young children. The costs of scaling up the required training through the integrated community case management platform in an average district, and of establishing emergency transport systems in areas of greatest need, are relatively modest (48 400 United States dollars per district; Box 3) when the likely improvements in health outcomes are considered. The reduction in reported deaths that occurred across our project districts suggests that rectal artesunate can be used effectively in real-world conditions with appropriate training of volunteers, including vital components on community mobilization and engagement.

Box 3 **Cost of scaling up use of rectal artesunate for severe malaria, Zambia, 2018–2021**

***Training***

2-day training in rectal artesunate administration and community mobilization (delivered as part of integrated community case management training), costing 12 400 United States dollars (US$) per district.

***Emergency transport system***

Scoping exercise to identify priority sites, 20 emergency transport system vehicles per priority district (including spare parts for vehicles) and training of 60 riders (including protective and safety items for riders), costing US$ 36 000 per district.

***Assumptions***

- An average district population of 150 000, 70% of whom are target rural population;
- the availability of a core group of trainers (there exist approximately 20 rectal artesunate trainers in Zambia as of 2022); and
- that volunteers and riders are supported by health facility staff who are, in turn, supported by a district health management team.
